# Early human B cell signatures of the primary antibody response to mRNA vaccination

**DOI:** 10.1073/pnas.2204607119

**Published:** 2022-06-27

**Authors:** Lela Kardava, Nicholas Rachmaninoff, William W. Lau, Clarisa M. Buckner, Krittin Trihemasava, Jana Blazkova, Felipe Lopes de Assis, Wei Wang, Xiaozhen Zhang, Yimeng Wang, Chi-I Chiang, Sandeep Narpala, Genevieve E. McCormack, Can Liu, Catherine A. Seamon, Michael C. Sneller, Sarah O’Connell, Yuxing Li, Adrian B. McDermott, Tae-Wook Chun, Anthony S. Fauci, John S. Tsang, Susan Moir

**Affiliations:** ^a^Laboratory of Immunoregulation, National Institute of Allergy and Infectious Diseases, National Institutes of Health, Bethesda, MD 20892;; ^b^Multiscale Systems Biology Section, Laboratory of Immune System Biology, National Institute of Allergy and Infectious Diseases, National Institutes of Health, Bethesda, MD 20892;; ^c^Institute for Bioscience and Biotechnology Research, Rockville, MD 20850;; ^d^Vaccine Research Center, National Institute of Allergy and Infectious Diseases, National Institutes of Health, Bethesda, MD 20892;; ^e^Critical Care Medicine Department, National Institutes of Health Clinical Center, National Institutes of Health, Bethesda, MD 20892;; ^f^Department of Microbiology and Immunology and Center for Biomolecular Therapeutics, University of Maryland School of Medicine, Baltimore, MD 21201;; ^g^NIH Center for Human Immunology, National Institute of Allergy and Infectious Diseases, National Institutes of Health, Bethesda, MD 20892

**Keywords:** mRNA vaccines, B cells, antibodies, adaptive immunity, SARS-CoV-2

## Abstract

Messenger RNA (mRNA) vaccines against severe acute respiratory syndrome coronavirus 2 (SARS-CoV-2) are highly effective at inducing protective immunity. However, weak antibody responses are seen in some individuals, and cellular correlates of immunity remain poorly defined, especially for B cells. Here we used unbiased approaches to longitudinally dissect primary antibody, plasmablast, and memory B cell (MBC) responses to the two-dose mRNA-1273 vaccine in SARS-CoV-2–naive adults. Coordinated immunoglobulin A (IgA) and IgG antibody responses were preceded by bursts of spike-specific plasmablasts after both doses but earlier and more intensely after dose 2. While antibody and B cell cellular responses were generally robust, they also varied within the cohort and decreased over time after a dose-2 peak. Both antigen-nonspecific postvaccination plasmablast frequency after dose 1 and their spike-specific counterparts early after dose 2 correlated with subsequent antibody levels. This correlation between early plasmablasts and antibodies remained for titers measured at 6 months after vaccination. Several distinct antigen-specific MBC populations emerged postvaccination with varying kinetics, including two MBC populations that correlated with 2- and 6-month antibody titers. Both were IgG-expressing MBCs: one less mature, appearing as a correlate after the first dose, while the other MBC correlate showed a more mature and resting phenotype, emerging as a correlate later after dose 2. This latter MBC was also a major contributor to the sustained spike-specific MBC response observed at month 6. Thus, these plasmablasts and MBCs that emerged after both the first and second doses with distinct kinetics are potential determinants of the magnitude and durability of antibodies in response to mRNA-based vaccination.

## Significance

The pandemic caused by severe acute respiratory syndrome coronavirus 2 (SARS-CoV-2) accelerated development of messenger RNA (mRNA) vaccines, which have proven to be highly effective against COVID-19. However, antibody responses vary widely and wane over time. This study evaluated the range and kinetics of the primary antibody response to SARS-CoV-2 mRNA-based vaccination in parallel with the B cells that are involved in generating and maintaining this response. These include plasmablasts, the antibody-secreting cells that arise rapidly yet transiently following immunization, and memory B cells, a heterogeneous population that can provide long-lasting immunity. Our results show that the antibody response was tightly linked to early plasmablasts, while the cellular response was sustained by a distinct population of memory B cells.

The pandemic caused by severe acute respiratory syndrome coronavirus 2 (SARS-CoV-2) instigated rapid worldwide COVID-19 vaccine prioritization strategies. Several vaccine candidates were developed, including two vaccines (Moderna mRNA-1273 and the Pfizer/BioNTech BNT162b2), based on novel messenger RNA (mRNA) platforms (1). Both mRNA vaccines encode a stabilized ectodomain of the spike protein trimer (S-2P) derived from the Wuhan Hu-1 isolate (2). Two doses of mRNA vaccines have been shown to be highly protective and elicit strong antibody responses (3, 4), although poorer responses have also been seen in some individuals, such as older adults (5, 6) and transplant recipients (7, 8), raising the question of what determines antibody response levels and whether cellular correlates can be defined. Several studies have shown that SARS-CoV-2 mRNA vaccines can elicit a durable cellular response, including among B cells (reviewed in (9)), with memory B cells (MBCs) shown to correlate with the antibody response (10).

In the B cell compartment, one of the first detected responses in the blood after a primary immunization is a short transient burst around days 7–10 of plasmablasts (PBs) that are probably induced by the extrafollicular response and potentially responsible for the early serum antibodies to the immunogen, reviewed in (11). While it is unclear whether PBs are direct precursors of bone marrow–resident plasma cells that are the main source of circulating antibodies (12), several studies on inactivated and attenuated vaccines have shown that the PB response can predict the magnitude and longevity of protective antibodies (13–16). Among these predictors are PB responses that are independent of antigen specificity (17, 18), suggesting that the quantitative extent of antigen-specific responses is coupled to that of the total PB responses detectable in blood, including bystander and PBs with weak affinity for detection (13, 15). For mRNA-based SARS-CoV-2 vaccines, several studies have described a robust yet highly variable PB response in blood and draining lymph nodes (5, 19), and there is evidence of a clonal relationship between PBs in the blood and MBCs in the lymph nodes (20). Despite these advances, the role of PBs and other B cell populations in the induction and longevity of antibodies following mRNA-based vaccination and how they differ across individuals and potentially contribute to variability in antibody responses have not been fully assessed.

Here we performed parallel antibody and cellular assays on frequent blood collections to capture the early events of the primary B cell response to the mRNA vaccine mRNA-1273. Using an unbiased approach, we identified PBs and other early B cell populations as correlates of the antibody response to SARS-CoV-2 mRNA-based vaccination.

## Results

### Robust yet Variable Antibody and Early B Cell Response.

**Fig. 1.**
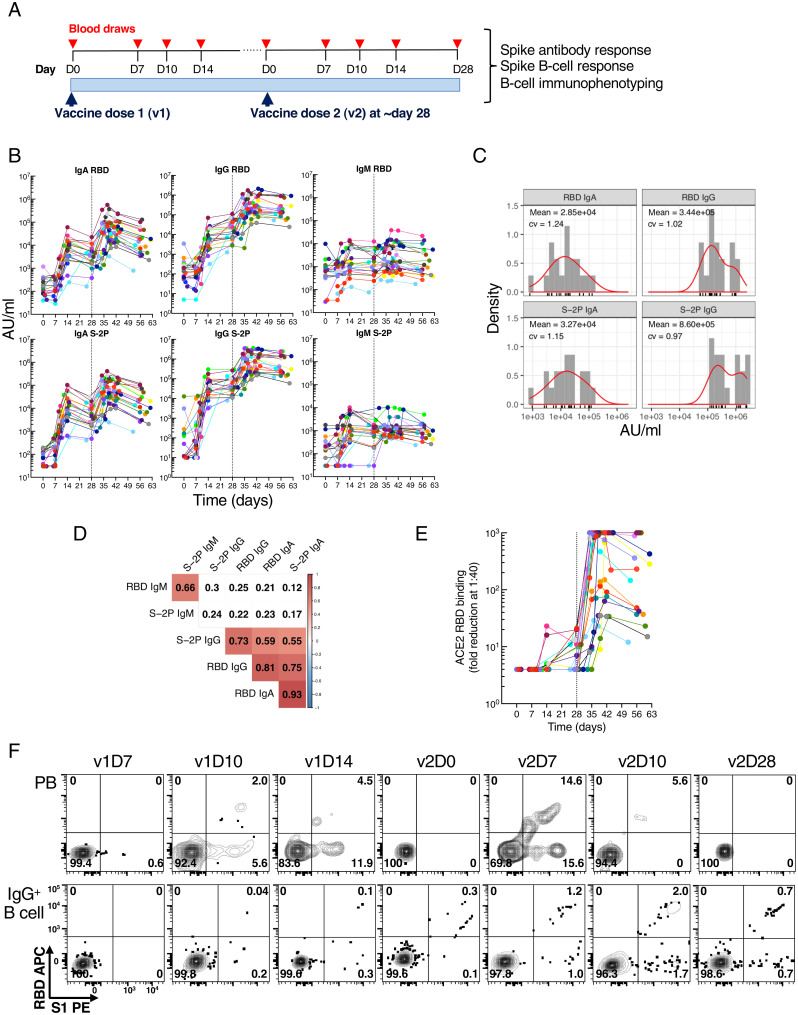
Longitudinal blood sampling and analysis shows robust antibody and early B cell response to mRNA-1273 vaccine. (*A*) Study design with serial blood draws and assays performed at all timepoints on SARS-CoV-2–uninfected vaccinees (*n* = 21; missed visits and exact timepoints in *SI Appendix*, Table S1) receiving two doses of the mRNA-1273 vaccine. (*B*) Serum IgG, IgA, and IgM binding to S-2P and RBD proteins measured by ECLIA longitudinally, and (*C*), corresponding histogram and distribution (based on kernel density estimates) at the last timepoint (v2D28). (*D*) Triangular heatmap of Spearman’s rank correlation between serum antibodies at last measured timepoint (v2D28) in (*B*). Numbers represent *r* values. Statistically insignificant correlations (*P* > 0.05) shown in white. (*E*) Longitudinal inhibition of RBD binding to ACE2 by serum (1:40 dilution) of vaccinees (*n* = 21). (*F*) Longitudinal binding of S1 and RBD tetramers to PB and IgG^+^ B cells by flow cytometry shown for a high responder (VAC-611; *SI Appendix*, Table S1). Numbers in each quadrant are percentages. Each vaccinee is color-coded, and second vaccine dose is indicated by vertical dotted line (*B* and *E*). AU, arbitrary units.

To longitudinally track the primary antibody and corresponding B cell response to the two-dose mRNA-1273 vaccine, we obtained blood from 21 healthy SARS-CoV-2–uninfected adults at several defined timepoints (up to nine) over a period of ∼60 d (Fig. 1*A* and *SI Appendix*, Table S1). Paired antibody and cellular assays were performed on each visit day (D), beginning at baseline (D0) of each dose, referred to henceforth as v1 and v2. Given the fragility of PBs, cellular assays were performed on freshly isolated cells while sera were cryopreserved for antibody assays. Antibody binding to S-2P and its receptor-binding domain (RBD) was measured for immunoglobulin G (IgG), IgA, and IgM on a multiplex platform (2). Strong IgG and IgA responses were induced, starting around D10, to both S-2P and RBD (Fig. 1*B*), although the magnitude was highly variable across vaccinees at v2D28 (c.v. > 100%), spanning two to three orders of magnitude for both IgA and IgG titers (Fig. 1*C*). The IgM response was weak across all vaccinees (Fig. 1*B*), consistent with reported differences between infection and immunization with mRNA vaccines (21). Strong correlations were observed between RBD and S-2P antibodies (Fig. 1*D*), with the correlation between IgA RBD and IgA S-2P being higher than that between their IgG counterparts. The inhibition of RBD binding to the spike protein receptor ACE2 by serum antibodies, a good surrogate for neutralization capacity (22), also revealed a range of responses (Fig. 1*E*) that correlated with RBD IgG and IgA binding antibodies (*SI Appendix*, Fig. S1*A*).

While flow cytometry has been widely used to detect SARS-CoV-2 spike-specific B cells following infection and vaccination (23–26), antigen-specific PBs are generally tracked separately by enzyme-linked immunospot (ELISpot) (19). We designed an approach with a pair of RBD and spike subunit 1 (S1) tetramers to simultaneously track spike-specific B cells and PB responses of vaccinees by spectral flow cytometry. Dual RBD^+^S1^+^ and single S1^+^ PB became detectable at v1D10, while spike-specific non-PB B cells became clearly detectable at v1D14 (Fig. 1*F* and *SI Appendix*, Fig. S1*B*). The combination of RBD and S1 tetramers also provided a strong indication of specificity both for PB and non-PB B cells, as evidenced by the rarity of RBD^+^ cells in the absence of S1 binding (Fig. 1*F* and *SI Appendix*, Fig. S1*C*). S-2P tetramers also clearly detected RBD^+^ within S1^+^ and S1^+^ within S-2P^+^ B cells (*SI Appendix*, Fig. S1*C*), as well as S-2P^+^ PB by ELISpot (*SI Appendix*, Fig. S1*D*), but they did not clearly identify S-2P^+^ PB by flow cytometry (*SI Appendix*, Fig. S1*C*). We thus focused on RBD and S1 tetramers to simultaneously measure spike-specific responses among all B cell populations. We validated our approach for assessing PBs by showing that 1) the frequencies of RBD^+^ and S1^+^ PB measured by flow cytometry were strongly correlated to those measured by the ELISpot assay (*SI Appendix*, Fig. S1*D*); 2) the distribution of IgG, IgA, and IgM among PBs was largely similar in the presence or absence of permeabilization (*SI Appendix*, Fig. S1*E*); and 3) IgG PB could clearly be visualized without the need for permeabilization, as is required by other methods (27), and clearly delineated from other isotypes (*SI Appendix*, Fig. S2*A*). On average, over 95% of all cells within the PB gate (CD3^−^CD19^+^CD20^−^CD27^++^CD38^++^; *SI Appendix*, Fig. S2*A*) had a detectable isotype, either IgA, IgG, or IgM (*SI Appendix*, Fig. S1*E*), indicating that spectral flow cytometry can be used to identify PBs and antigen-specific PBs without the need for permeabilization.

### Unbiased Cell Clustering Analyses Reveal Intense PB and MBC Response Early after Dose 2.

**Fig. 2.**
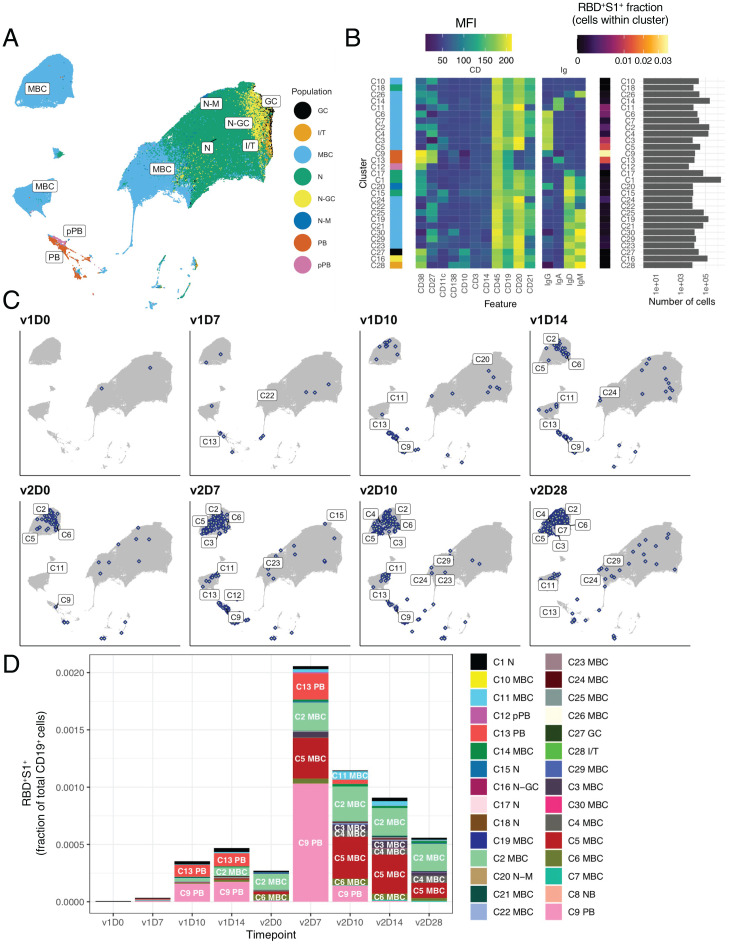
Unsupervised clustering analysis identifies major B cell populations and SARS-CoV-2–specific B cells. (*A*) UMAP projection of combined B cells (*n* = 653,683 cells), subsampled from 3.2 million CD19^+^ cells to include 3,667 cells per sample and all RBD^+^S1^+^ cells from all study participants (*n* = 21) at all timepoints with annotated major B cell populations identified by FlowSOM clustering. (*B*) MFI-based heatmap of FlowSOM clusters as indicated by cluster number and marker. Rows ordered by hierarchical clustering. Summary of fraction of cells binding both RBD and S1 within each cluster and cell counts per cluster (*Right*). (*C*) UMAP plots with overlays of RBD^+^S1^+^ B cells (blue points with white center) at each timepoint. (*D*) RBD^+^S1^+^ cells within each cluster expressed as a fraction of total CD19^+^ B cells across all subjects at each timepoint (*n* at each timepoint shown in *SI Appendix*, Table S1).I/T, immature transitional; N, naive; pPB, preplasmablast.

B cells that circulate in the peripheral blood are highly heterogeneous (28), especially MBCs, which undergo phenotypic and functional changes over time following antigen exposure (29). To capture a broad spectrum of phenotypes, including the four major immunoglobulin isotypes, we designed a spectral flow cytometric panel that combined 15 antibodies against primarily B cell markers and two spike tetramers (*SI Appendix*, Table S2). Unsupervised clustering analysis of the 15 cell surface markers on CD19^+^ single B cells, performed on all vaccinees and all timepoints simultaneously, revealed 30 clusters grouped by eight major B cell populations, including naive, PB, and MBC subsets (Fig. 2*A*). A more granular depiction of individual clusters (*SI Appendix*, Fig. S2*B*), with corresponding gating strategy (*SI Appendix*, Fig. S2*A*) and annotations (*SI Appendix*, Table S3), demonstrated the heterogeneity of the B cell populations observed. A corresponding mean fluorescence intensity (MFI) heatmap delineated the surface marker phenotype, isotype, and specificity of individual cell clusters (Fig. 2*B*) and identified naive B cells (C1) as the most abundant cluster, as expected for peripheral blood (27). Five populations of conventional (CD27^+^CD20^+^CD21^+^) MBCs, each distinguished by isotype (IgA^+^ [C14], IgG^+^ [C2 and C4], and IgD/M [C19]), were prevalent (Fig. 2*B*). C2 and C4 differed by expression of CD38, a marker that was the distinguishing feature between several of the MBC clusters (Fig. 2*B* and *SI Appendix*, Table S3), consistent with its role as a marker for delineating various human B cell populations (28). Both IgG (C9) and IgA (C13) PBs were identified, as well as eight additional MBCs, collectively defined as nonconventional (*SI Appendix*, Table S3), with lower abundance (Fig. 2*B*). IgG PB C9 contained the highest proportion of RBD^+^S1^+^ cells, followed by IgA PB C13 and the nonconventional IgG^+^ MBC C5 (Fig. 2*B*). The phenotype of C5, CD27^+^CD20^++^CD21^lo^CD11c^+^, is consistent with antigen-activated B cells that have been reported by others following SARS-CoV-2 infection or vaccination (26, 30–32). Longitudinal tracking of the spike-specific response within clusters revealed that RBD^+^S1^+^ PBs were first detected at v1D10, then subsided until v2D5–7, while other RBD^+^S1^+^ B cells (namely MBCs) became visible at v1D14 and intensified significantly following v2 (Fig. 2 *C* and *D*), consistent with the analyses performed with manual gating (Fig. 1*F* and *SI Appendix*, Fig. S1*B*).

### Kinetics of Antigen-Nonspecific and -Specific B Cells in Response to Vaccination.

**Fig. 3.**
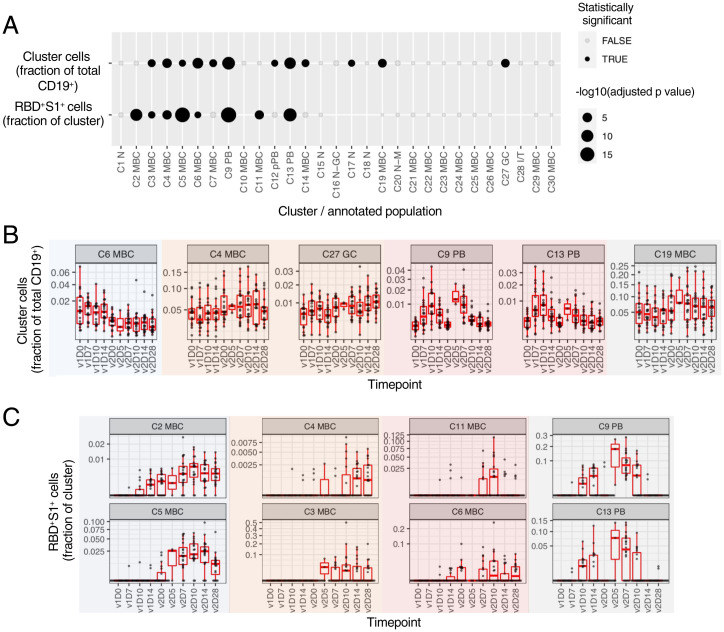
Antigen-nonspecific and spike-specific cells exhibit temporal change in response to the mRNA-1273 vaccine. (*A*) Clusters showing significant temporal variation over course of v1 and v2 in the frequency of nonspecific cells as a fraction of total (CD19^+^) B cells (first row) and RBD^+^S1^+^ cells as a fraction within each cluster (second row). (*B*) Longitudinal display of nonspecific cells per cluster as a fraction of total (CD19^+^) B cells, shown for clusters with statistically significant temporal variations, as shown in (*A*). Clusters were grouped by temporal patterns (see *Methods*); groups are shown with background colors. Lines denote the mean, and shading denotes 95% bootstrap confidence interval per timepoint. Values rescaled as fraction of maximum 95% confidence interval estimate over the entire time course. (*C*) similar to (*B*) but displaying of RBD^+^S1^+^ cells as a fraction within each cluster. Type III ANOVA test using Satterthwaite’s approximation (*A*). N at each timepoint shown in *SI Appendix*, Table S1 (*B–E*).

We next used a linear model to search for cell clusters (both antigen-nonspecified, referred to as “nonspecific” henceforth, and “specific” otherwise) whose frequencies varied significantly as a function of time in response to vaccination (Fig. 3*A*). Antigen-nonspecific clusters exhibited distinct patterns of response kinetics, including most notably sharp increases in the two PBs, IgG C9 and IgA C13, after v1 and v2 (Fig. 3*B*). Other temporally changing clusters included several MBCs and CD10^+^CD27^+^ germinal center (GC) founder B cells (C27) that underwent modest changes after v1 and v2 (Fig. 3*B*), possibly a reflection of trafficking to and from lymphoid tissues (33).

Spike-specific B cell frequencies, measured as a fraction of RBD^+^S1^+^ cells among cells within each cluster, also exhibited varying patterns of temporal responses (Fig. 3*C*). Two-peak responses with stronger increases in v2 than v1 were observed for the two PB clusters C9 and C13, nonconventional MBC C11 (CD27^lo^IgA^+^), and C6 (CD27^−^IgG^+^), albeit with differences in timing. For example, C9 (IgG) and C13 (IgA) PB had an initial modest burst beginning at v1D10, followed by a second stronger but shorter burst at v2D5–7 (Fig. 3*C*). RBD^+^S1^+^ cells among two pairs of conventional/nonconventional MBCs, namely C2/C5 and C4/C3, respectively, also underwent coordinated changes following v2 (Fig. 3*C*). It is notable, however, that while RBD^+^S1^+^ cells in all four of these MBC clusters showed trends of declines from their peaks by v2D28, those in the two nonconventional CD21^lo^CD11c^+^ MBCs (CD27^−^ C3 and CD27^+^ C5) appeared to drop more precipitously than in the two conventional CD27^+^CD21^+^ MBCs (CD38^+^ C2 and CD38^−^ C4; Fig. 3*C*). These findings are consistent with recent reports that spike-specific memory responses several months after SARS-CoV-2 infection or vaccination are enriched within resting MBCs (10, 26, 31), which have a phenotype similar to C2 and C4.

Despite the qualitively coherent changes observed across subjects, substantial heterogeneity in B cell responses existed among vaccinees. Independent of antigen specificity, the magnitude of PB increases is known to be a correlate of antibody responses for vaccines such as influenza (17, 18). We thus assessed associations between the changes in nonspecific cluster frequencies over the course of v1 and v2 relative to the respective baselines with IgA and IgG RBD or S-2P titers at v2D28 by using linear models accounting for age and sex (*SI Appendix*, Fig. S3 *A* and *B*). Both IgA and IgG PBs (C9 and C13) at v1D10 were indeed positively associated, albeit mildly, with both RBD and S-2P IgA titers (*SI Appendix*, Fig. S3 *A*, *C*, and *D*), while several MBC clusters at as early as v1D7 (C3, C11, C24) and v1D10 (C6, C7) were correlated with S-2P IgG titers (*SI Appendix*, Fig. S3*A*).

### Spike-Specific PBs and MBCs Correlate with Antibody Response.

**Fig. 4.**
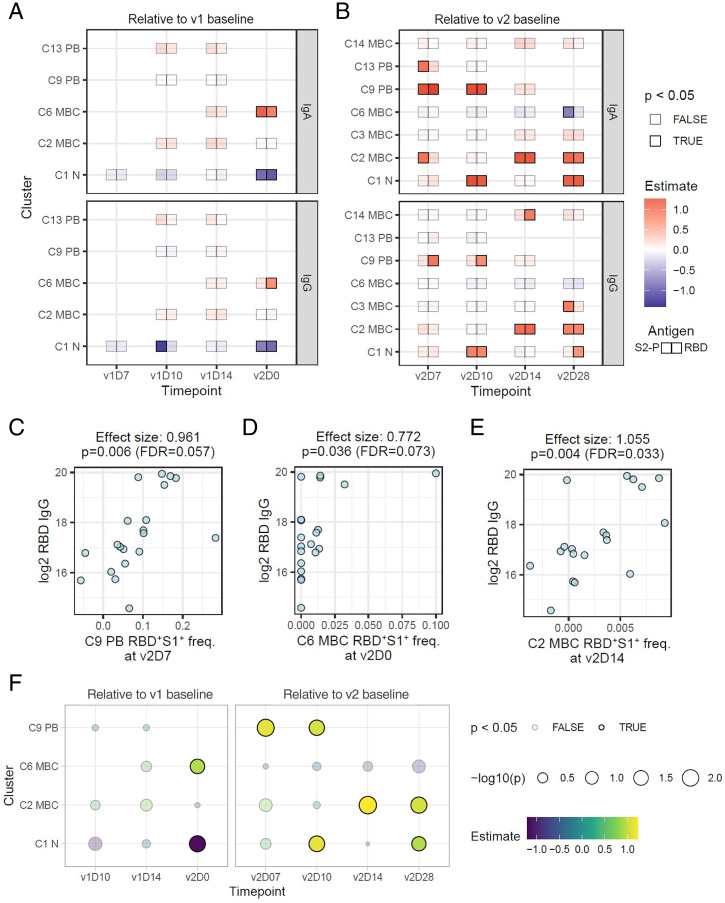
Correlates of SARS-CoV-2 antibody titers 28 d after second dose of vaccine. (*A* and *B*) Linear model effect size estimates indicate strength of association between spike-specific (RBD^+^S1^+^) cell frequency in the cell clusters (rows) with antibody endpoints (IgA and IgG titers for S-2P and RBD) relative to prevaccination baseline level (v1D0) at four timepoints between v1 and v2 (*A*) and relative to v2 baseline (v2D0) at four timepoints after v2 (*B*). Only clusters with at least one significant (unadjusted *P* ≤ 0.05) association at any timepoint are shown. At each timepoint, clusters that had fewer than five samples with any RBD^+^S1^+^ cells were excluded from analysis (missing boxes). (*C–E*) Scatter plots illustrating correlations between endpoint (v2D28) RBD IgG titers and RBD^+^S1^+^ cell frequencies in C9 on v2D7, C6 on v2D0, and C2 on v2D14, respectively. Effect sizes and *P* values were estimated by the linear models above. FDR estimate of the statistical significance was calculated within each antibody endpoint and timepoint combination. (*F*) Effect size estimates of association between first principal component (PC1) of endpoint SARS-CoV-2 antibody titers and spike-specific RBD^+^S1^+^ (double positive) cell frequencies within each cell cluster. PC1 was derived from IgA and IgG titers against S-2P and RBD proteins at v2D28. Only cell clusters with at least one significant (unadjusted *P* ≤ 0.05) association at any timepoint are shown. FDR, false discovery rate; ρ, Spearman’s rank correlation.

The frequency of spike-specific PBs on v2D7 spanned a wide range (Fig. 3*C*), raising the question of whether those with depressed PB responses also had lower antibody titers following v2. Thus, we next used the same linear model to search for spike-specific correlates (Fig. 4 *A* and *B*). Indeed, at v2D7 and v2D10, IgG PBs (C9) were correlated with IgG and IgA RBD antibodies, while IgA PBs (C13) at v2D7 were associated with IgA S-2P antibodies (Fig. 4 *B* and *C*). A recent study did not find a correlation between antibody response and transcriptional modules enriched for PBs at D7 following the second dose of the Pfizer vaccine (34), probably due to differences in assessing PB responses by using blood transcriptional signatures versus our direct measurement of fresh, antigen-specific PBs. In addition to PBs, spike-specific C6 was a positive correlate at v2D0 (Fig. 4 *A* and *D*) when these cells reached a first peak (Fig. 3*C*). C6 is a population of CD27^−^IgG^+^ MBCs known to have lower mutational burdens than their CD27-expressing counterparts (35) and as such may reflect products of early events of the antigen-driven maturation process after v1. Most of the other antigen-specific correlates were positively associated with the titer response and reflect changes after the second dose (Fig. 4*B*), including among MBCs. Notably at v2D14 and v2D28, the frequency of RBD^+^S1^+^ cells in the conventional CD27^+^CD21^+^ MBC C2 was positively correlated with RBD/S-2P IgA and IgG antibodies (Fig. 4 *B* and *E*), consistent with the role of these MBCs in sustaining immunological memory (26, 31).

Among the clusters that correlated with antibodies, several were not of the same isotype. While it is possible that IgG B cells could give rise to IgA-secreting cells, the reverse is rare (36). Thus, the most likely explanation for interisotype correlations is that most are not causal but reflect a coordinated immunologic response driven by shared mechanisms, as indicated by the strong correlations between IgG and IgA antibodies (Fig. 1*D*). Indeed, when we used the correlated component (the first principal component) of the v2D28 IgG and IgA RBD and S-2P antibody titers as an isotype-independent endpoint, we found many of the positive antigen-specific correlates highlighted above, including C6 MBC at v2D0, C9 PB at v2D7, and C2 MBC at v2D14 (Fig. 4*F*), indicating that these correlates reflected isotype-independent responses that potentially determined the magnitude of antibodies induced by the mRNA vaccine.

### Early B Cell Response Correlates with Late Antibody Titers.

**Fig. 5.**
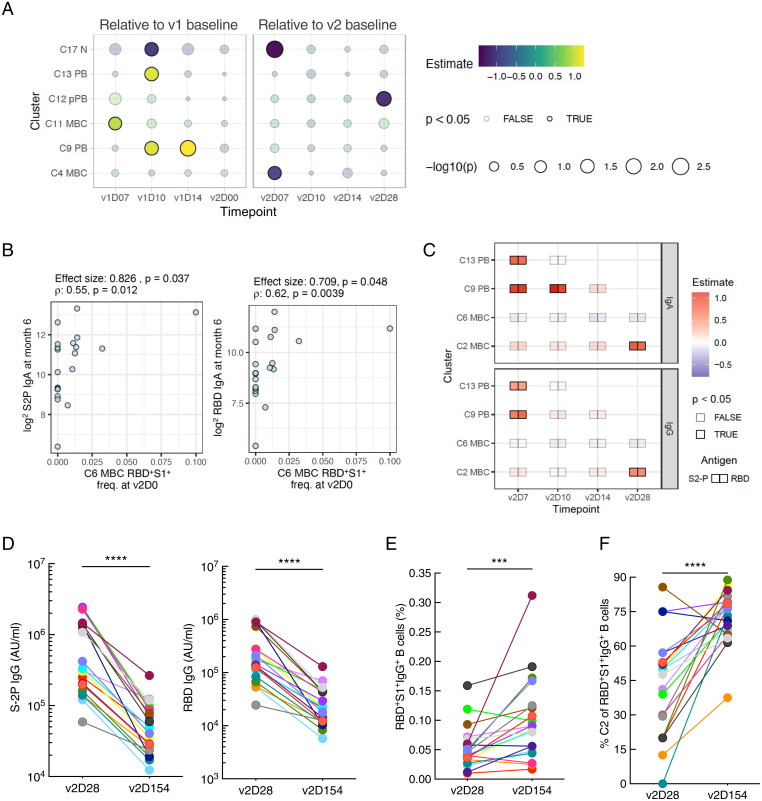
B cell correlates of antibody response at month 6. (*A*) Similar to Fig. 4*F*, associations between the first principal component (PC1, isotype independent) of antibody endpoint at month 6 (v2D154) and antigen-nonspecific cells. (*B*) Similar to Fig. 4*C*, scatter plots illustrating correlations between month-6 (v2D154) S-2P and RBD IgA titers and RBD^+^S1^+^ cell frequencies in C6 on v2D0. Shown are effect size estimated by linear model and Spearman’s correlation. (*C*) Similar to Fig. 4*A*, associations of month-6 (v2D154) antibody titers with spike-specific (RBD^+^S1^+^) cell frequency in the cell clusters at indicated timepoints. (*D–F*) Changes between v2D28 and v2D154 in vaccinees (*n* = 20), color-coordinated as in Fig. 1. (*D*) Serum IgG binding to S-2P and RBD proteins measured by ECLIA. (*E*) RBD^+^S1^+^ IgG^+^ B cell frequencies. (*F*) The proportion of total RBD^+^S1^+^ IgG^+^ B cells that are in C2. Wilcoxon signed rank test; ***P < 0.001; ****P < 0.0001 (*C–E*). AU, arbitrary units; ρ, Spearman’s rank correlation.

Among the 21 individuals originally recruited (*SI Appendix*, Table S1), 20 remained SARS-CoV-2 uninfected at month 6 and returned for an additional blood draw. First, we evaluated which of the early B cell correlates of the antibody response at v2D28 were still present several months later. Among nonspecific B cell correlates of the month-6 antibody response, these were assessed by using the isotype-independent antibody responses as the endpoint (i.e., first principal component of the v2D154 antibody titers, as described for Fig. 4*F*). We found several early positive cellular correlates of the v2D28 antibody response (*SI Appendix*, Fig S3*F*) were also correlated with antibody levels at v2D154. These included both IgG (C9) and IgA (C13) PBs at v1D10 and v1D14 for C9 (Fig. 5*A*). For antigen-specific responses that positively correlated with the month-6 IgG or IgA antibody titers, the earliest correlate was CD27^−^IgG^+^ MBC C6 at v2D0 (Fig. 5*B*), followed by IgG (C9) and IgA (C13) PBs at v2D7, C9 at v2D10, and CD27^+^IgG ^+^ MBC C2 at v2D28 (Fig. 5*C*). It is noteworthy that while IgG titers decreased sharply between months 2 (v2D28) and 6 (Fig. 5*D*), frequencies of spike-specific IgG^+^ B cells increased (Fig. 5*E*), consistent with a recent study (10). Furthermore, the CD27^+^IgG^+^ MBC C2 fraction of total RBD^+^S1^+^IgG^+^ B cells also increased, becoming the major contributor to the spike-specific IgG B cell response at month 6 (Fig. 5*F*) and suggesting that the sustained MBC response was driven by this population.

## Discussion

The antibody response elicited by mRNA vaccines against SARS-CoV-2 has been shown to be highly protective (3, 4), although with a high degree of heterogeneity (5–8, 37) and evidence of waning over time (38). By performing frequent, coordinated, and comprehensive interrogation of the primary antibody and B cell responses to the mRNA-1273 vaccine in a diverse cohort of naive individuals, we have identified several early B cell populations that correlated with the antibody response to this novel vaccine platform. By performing the analyses in real time via an integrated approach based on fresh cells and spectral flow cytometry, we have identified early PBs, both antigen nonspecific (within v1) and specific (early in v2), as strong correlates and potential determinants of the primary (month 2) and late (month 6) antibody response. The expansion of PBs in the acute phase of SARS-CoV-2 infection is well established (26, 32, 39) although often of undefined specificity and with unknown contribution to protection from reinfection. It is noteworthy that over 84% of PBs induced by dose 1 of the vaccine do not show reactivity to the SARS-CoV-2 spike proteins, possibly an indication of strong bystander or systemic immune activation (13, 15). However, timing may play a minor role as PBs are known to peak in blood within a narrow window not completely concomitant with our days 7, 10, and 14 sampling after dose 1. Moreover, our assays may not be sufficiently sensitive to have captured early specific PBs of low affinity or cross-reactivity with other coronaviruses, which may contribute to the early antibody response to SARS-CoV-2 (40). The v1 PB contrasted with the high specificity and more rapid induction of v2 PB (peaking at day 7), indicative of a strong recall response and persisting GC reaction (19).

A role for PBs in predicting the antibody response to SARS-CoV-2 vaccination has not been reported thus far. This is probably because of difficulties in integrating PBs into B cell panels for antigen-specific analyses, their transience in the blood following vaccination (29), compounded with the deleterious effects of cryopreservation on PB (27). Our real-time integrated analyses on frequently collected fresh cells have overcome these obstacles and provided an opportunity to reveal a clear role for PBs in the antibody response to mRNA-based vaccination. However, while the effect of early PBs on antibodies was consistently observed over the 6-month study period, the role of early antigen-specific and nonspecific PBs as potential drivers of the antibody response to primary mRNA-based vaccination will need to be validated with larger cohorts and different demographics. Nonetheless, our findings provide a roadmap for when and how to look for PB responses to mRNA vaccines.

Two antigen-specific MBCs were also found to correlate with the antibody response. C6, an IgG-switched MBC lacking expression of CD27 and previously shown to be less affinity matured than CD27-expressing counterparts (35), was an early v2 correlate (v2D0) of both the month-2 and month-6 antibody responses. C6 MBCs are also CD21^+^ and similar to a stable pool of CD27^−^ MBCs that are generated in response to new influenza variants (41). This MBC population may thus serve as both a precursor of more mature MBCs and a stable repository poised for rapid reactivation upon exposure to the pathogen. It is notable that C5, a nonconventional CD27^+^CD21^lo^CD11c^+^ MBC, was a major fraction of the RBD/S1-specific B cell response following v2, yet it did not correlate with the antibody response. C5 MBCs are similar to antigen-activated B cells that are thought to expand outside or independently of GC (42). They have been described in acute COVID-19 infection (32, 43) as well as chronic conditions such as HIV (44) and autoimmune diseases (45), where these B cells have been shown to express high levels of T helper 1 cell master transcription regulator, T-bet. In mice, a similar population of T-bet–expressing B cells may contribute to the clearance of pathogens (reviewed in (46)). However, it remains unclear what role C5 and similar T-bet-expressing MBCs play in humoral immunity following infection and vaccination in humans.

Finally, C2, an IgG-switched conventional (CD21^+^CD27^+^) MBC that has been associated with long-lasting B cell memory to SARS-CoV-2 vaccination and infection (26, 30), was found at v2D28 to correlate with both concurrent (v2D28) and month-6 antibody responses. In addition, C2 was the major contributor to the sustained B cell memory response that we and others have reported (10, 47). Given evidence that the MBC response may be broader than the antibody response (48), these MBCs may also be critical in preventing severe disease in breakthrough infections when antibody titers wane or antibody-resistant variants emerge. While not possible to address here, another question is whether the timing between vaccine doses might impact the quality, quantity, and longevity of spike-specific MBCs, possibly contributing to the enhanced antibody responses that have been reported when the interval between the two doses of mRNA vaccines was extended beyond 3–4 wk (49).

In conclusion, we used an integrated unbiased approach to provide a detailed view of the primary B cell response to SARS-CoV-2 mRNA-based vaccination. We identified PBs as early correlates of the antibody response at least up to 6 months. We also identified a distinct population of MBCs (C2) that both correlated with the antibody response and was a major contributor to the sustained cellular response out to at least month 6. These findings provide guidance and tools for future studies, either on different cohorts, including those of people with preexisting conditions, or different mRNA-based vaccines, and provide insight into the B cell populations involved in generating and maintaining protective immunity.

## Materials and Methods

### Study Design and Participants.

Twenty SARS-CoV-2–uninfected National Institutes of Health (NIH) employees and one community member who were eligible to receive an Emergency Use Authorization COVID-19 vaccine were recruited to study longitudinal vaccine responses. The 21 participants received a first dose of the Moderna mRNA-1273 vaccine, and 25–34 d later, 20 participants received a second dose of the same vaccine (*SI Appendix*, Table S1). One participant was lost to follow-up after testing positive for COVID-19 between doses. All remaining 20 participants had undetectable titers to the SARS-CoV-2 nucleocapsid protein (NP), as measured by the Bio-Rad Platelia assay, through 6 months after vaccination, except for one participant (VAC-005) whose NP titer predated the COVID-19 pandemic. An additional 11 participants who received both doses of the Moderna mRNA-1273 vaccine were recruited for the validation analyses of PB frequencies between flow cytometry and ELISpot; nine were SARS-CoV-2–uninfected and two had recovered from COVID-19 infection. All phlebotomy was performed at the NIH Clinical Research Center in Bethesda, MD under protocols approved by the NIH Institutional Review Board for research involving human subjects, ClinicalTrials.gov identifiers NCT00001281 and NCT04411147. NCT00001281 is a general blood draw protocol that allows for the enrollment of HIV-infected and HIV-uninfected individuals, the latter being the focus of the current study. All participants provided written informed consent.

### Blood Sample Collection and Processing.

Peripheral blood mononuclear cells (PBMCs) were isolated by Ficoll density gradient centrifugation from whole blood collected in ethylenediaminetetraacetic acid Vacutainer tubes. Serum was isolated by centrifugation of clotted whole blood collected in serum separation transport Vacutainer tubes and stored at –80 °C.

### SARS-CoV-2–Binding Antibody Assay.

Serum was heat inactivated at 56 °C for 60 min. A 4-plex antibody binding assay was performed with an electrochemiluminescence immunoassay analyzer (ECLIA) developed by Meso Scale Discovery (MSD). Each well of MSD SECTOR plates was precoated by the manufacturer (MSD) with SARS-CoV-2 spike (S-2P), RBD protein, nucleocapsid protein, and a bovine serum albumin (BSA) in a specific spot designation for each antigen. Plates were blocked at room temperature (RT) for 60 min with MSD blocker A solution containing 5% BSA. Plates were washed and MSD reference standard (calibrator), QC test sample (pool of COVID-19 convalescent sera), and human serum test samples were added in duplicate in an eight-point dilution series, and reference standards were added in triplicate. MSD Control sera (low, medium, and high) were added undiluted in triplicate. Samples were incubated with shaking at RT for 4 h on a Titramax Plate Shaker (Heidolph). Plates were washed and incubated with MSD SULFO-TAG anti-human IgG, IgA, or IgM detection antibodies at RT for 60 min with shaking. Plates were washed, MSD GOLD read buffer containing electrochemiluminescence (ECL) substrate was added, and plates were read with the MSD MESO Sector S 600 detection system. A similar 10-plex antibody binding assay, previously described (50), was performed to detect v1D0 antibody titers to the spike proteins of SARS-CoV and circulating alpha (229E, NL63) and beta (HKU1, OC43) coronaviruses. Analyses were performed with Excel (Microsoft) and Prism 9.0 (Graphpad) software, and antibody concentrations were assigned arbitrary units (AU/mL) by interpolation from the standard curve. Densities of antibody concentrations at endpoint (v2D28) were estimated via a Gaussian kernel with bandwidth automatically selected through biased cross-validation by the stat_density function from ggplot2 (3.3.3) with bw = “bcv”.

### RBD-ACE2 Blocking Assay.

Samples were prepared as for the 4-plex binding assay. The 384-well plates precoated with RBD were supplied by the manufacturer (MSD). Plates were blocked at RT for 30 min with MSD blocker A solution containing 5% BSA. Plates were washed, test samples were added at dilutions of 1:10, 1:20, and 1:40, and plates were incubated with shaking at RT for 60 min. Human ACE2 conjugated with SULFO-TAG was added, and plates were further incubated to allow binding to RBD. Plates were washed, ECL substrate added, and plates read as in the antibody binding assay. Fold reduction in ECL response for each sample was calculated based on signal emitted in wells in the absence of sample (assay diluent).

### Recombinant Biotinylated RBD Protein.

A SARS-CoV-2 RBD construct containing a His-tag and Avi-tag was generated, as previously described (51). Residues 319–541 of the S protein were codon optimized with the N-terminal of signal peptide (MFVFLVLLPLVSSQ) and C-terminal of 6-His tag and Avi-tag (GLNDIFEAQKIEWHE). The DNA encoding sequence was cloned into the mammalian cell expression vector pCAGGS and confirmed by sequencing, before transient transfection in FreeStyle 293-F cells with 293fectin transfection reagent (Thermo Fisher). Culture supernatants were harvested at 5 d after transfection, filtered, and purified by in-house packed affinity purification column with cOmplete His-tag purification resin (Roche). Elutes were buffer exchanged with phosphate-buffered saline (PBS) and concentrated with an Amicon Ultra 10 kDa molecular weight cutoff concentrator (Millipore). Biotinylation was performed with a BirA biotin-protein ligase standard reaction kit (Avidity), according to the manufacturer’s instructions. Excess biotin was removed by five buffer exchanges with an Ultra 10K concentrator (Amicon).

### B Cell Spike-Specific Responses and Phenotyping by Flow Cytometry.

A 17-color panel was developed to phenotype B cell populations and identify SARS-CoV-2–specific B cells among PBMCs by spectral flow cytometry (*SI Appendix*, Table S2 for list and source of antibodies and biotinylated spike proteins). The biotinylated spike proteins were tetramerized with fluorescently labeled streptavidin (SA) as follows: S1 with SA-R-phycoerythrin (PE), RBD with SA-allophycocyanin (APC), and S-2P with SA-Alexa Fluor 488 (Thermo Fisher Scientific). In a stepwise process, 1/5 of the molar equivalent of the SA-fluorochrome reagent was added to the biotinylated protein at 20-min intervals until the molar ratio of biotinylated protein and SA-fluorochrome reached 4:1. Incubations were carried out at 4 °C with gentle rocking. To titrate the labeled protein tetramers and establish background and antigen specificity, freshly isolated or cryopreserved PBMCs from SARS-CoV-2 uninfected and recovered infected individuals were used as negative and positive controls, respectively. For vaccinees, 10^6^ freshly isolated PBMCs were stained with a mixture containing the 15 panel antibodies and 160 ng each of PE-conjugated S1 and APC-conjugated RBD in staining buffer (2% fetal bovine serum [FBS]/PBS) supplemented with Brilliant Stain Buffer Plus (BD Biosciences) at 4 °C for 30 min. In a second 18-color panel, 400 ng of Alexa Fluor 488 conjugated S-2P was added to the mixture. The stained cells were acquired on an Aurora spectral cytometer in SpectroFlo Software v2.2.0 (Cytek Biosciences) and analyzed in FlowJo v10.7.1 (BD Biosciences).

### Intracellular Flow Cytometry.

PBMCs were first stained with fluorophore-labeled antibodies against cell surface markers CD3 (OKT3, BioLegend), CD19 (SJ25-C1, Thermo Fisher), CD20 (*SI Appendix*, Table S2), and CD27 (O323, BioLegend), fixed (Lysing Solution, BD Biosciences), permeabilized (Permeabilizing Solution 2, BD Biosciences), and stained with fluorophore-labeled antibodies against IgG (G18-145, BD Biosciences), IgA (8E10, Miltenyi Biotec), IgD (IA6-2, BioLegend), and IgM (MHM-88, BioLegend). The stained cells were acquired on a FACS Canto II flow cytometer (BD Biosciences) and analyzed in FlowJo software v9.9.6 (BD Biosciences).

### ELISpot Assay to Enumerate SARS-CoV-2 Spike-Specific PBs.

Spike protein S1 and RBD-specific antibody-secreting PBs were enumerated by modifying the antigen-specific portion of a previously described ELISpot assay (52, 53). Briefly, wells of Immobilon-P polyvinylidene difluoride membrane 96-well plates (Millipore) were coated with 5 μg/mL anti-Ig light-chain antibodies (Rockland Immunochemicals) overnight at 4 °C. Plates were washed and wells were blocked at RT for 2 h with RPMI medium containing 10% FBS. Duplicate wells were plated with PBMCs containing 0.01–0.003 × 10^6^ B cells for total IgA/G/M-secreting PB enumeration and 0.1–0.03 × 10^6^ B cells for RBD/S1-specific PBs. Plates were incubated at 37 °C for 5 h, followed by overnight incubation at 4 °C with biotinylated antibodies (Jackson Immunoresearch) against IgA (catalog no. 109-066-011), IgG (catalog 709-066-149), IgM (catalog no. 709-066-073), or biotinylated proteins S1 (ACROBiosystems, catalog no. S1N-C82E8) or RBD (BioLegend, catalog no. 790904). Plates were washed and streptavidin-AP conjugate (R&D Systems) was added, followed by incubation at RT for 2 h. Plates were washed and spots were developed with ELISpot Blue Color Module (R&D Systems). Biotinylated (Biorbyt) or unlabeled (Millipore-Sigma) keyhole limpet hemocyanin (KLH) was used as negative control antigen to enumerate background spots. Spots were counted with an ELISPOT reader (Cellular Technology Ltd.). Frequencies of S1 and RBD-specific PBs were calculated as the fraction of total Ig-secreting PBs after subtraction of background KLH spots.

### Spectral Flow Cytometry Data Processing for FlowSOM Clustering and UMAP Embedding.

FCS files generated from spectral flow cytometry with the 17-color panel and associated manual gates were read into R via FlowWorkspace (4.2.0). Live, CD19^+^ single cells were selected for downstream analysis. FlowSOM clustering was performed on the unscaled intensity values without z-scoring or additional transformation. The following markers were used to perform clustering CD20, CD138, CD38, CD10, CD11c, CD19, CD27, CD21, IgD, IgM, IgG, IgA, in FlowSOM (1.22.0) (54), with the number of desired metaclusters (nClus) set to 30. One small cluster, C8, determined to represent granulocytes, was removed from downstream analysis. To visualize the clusters and RBD^+^S1^+^ cells in a uniform manifold approximation and projection (UMAP) embedding (55), 653,683 cells were subsampled from the roughly 3.2 million CD19^+^ cells to include 3,667 cells per sample and all RBD^+^S1^+^ cells from the 21 longitudinal vaccine participants at all timepoints. Data were transformed as described above, and the UMAP embedding was fit in the uwot package (0.1.10) with default parameters.

### Analysis of Temporally Varying FlowSOM Clusters.

Frequencies of cells within each sample were summarized into 1) the fraction of cells within a cluster relative to the total number of CD19^+^ cells in the given sample and 2) the fraction of cells within a cluster determined to be RBD^+^S1^+^ by manual gating, relative to the number of cells in that cluster in the given sample. The extent of temporal variation was assessed via a linear mixed effects model with the following formula in lme4 (1.1.26):

Frequency∼timepoint+(1|Subject_ID)

Timepoint is a factor variable representing the discrete timepoints (v1D0, v1D7, etc.).

The significance of the timepoint term was assessed with a type III ANOVA via Satterthwaite’s approximation in v3.1.3 of the lmerTest package (56). *P* values were adjusted across all comparisons via the Benjamini–Hochberg procedure (57). Clusters with adjusted *P* values below 0.05 were deemed temporally fluctuating. Clusters selected by the above procedure were then grouped by the similarity of their temporal patterns. Briefly, the mean frequency across all subjects at a given timepoint was computed along with the 95% bootstrap confidence intervals around the mean in package Hmisc (v4.5.0). The means were then rescaled by dividing all values by the maximum value of the 95% confidence interval throughout the time-course such that all values were now in the range of [0,1]. The clusters were then grouped by hierarchical clustering of the mean trends using the Euclidean distance at each timepoint and using Ward’s method, as implemented in the hclust function (method = “ward.D2) in R (v4.0.2). After the respective dendrograms were inspected, four groups were determined to be appropriate, and the hierarchical clustering trees were cut to produce four groups for both antigen-nonspecific and -specific cells.

### Modeling of Association between Endpoint Antibody Concentrations and Cluster Frequencies.

A linear model accounting for the age and sex of the longitudinal vaccine participants was used to estimate whether cell cluster frequencies in response to vaccination were associated with SARS-COV2 spike protein (S-2P/RBD) antibody concentration at endpoint (v2D28):

$$\text{log2}\left(\text{endpoint}\;\text{concentration}\right)∼\text{cluster}\;{\text{frequency}}^{\text{timepoint}}+\text{age}+\text{sex}$$

Analyses were carried out on both standardized antigen-nonspecific and -specific frequencies (i.e., cluster cell counts as a fraction of total CD19^+^ cell counts and RBD^+^S1^+^ cells within the clusters, respectively). For the antigen-nonspecific models, only clusters whose postvaccination frequencies at any timepoint changed significantly from the prevaccination baseline (v1D0) were included. For the antigen-specific models, clusters with at least four RBD^+^S1^+^ cells in any of the samples were considered. In addition, at each timepoint, a cluster was excluded if there were fewer than five samples with any RBD^+^S1^+^ cells. Prevaccination baseline frequencies (v1D0) were subtracted from frequencies after vaccine dose 1 (v1) timepoints, including v1D7, v1D10, v1D14, and v2D0. Similarly, dose 2 baseline frequencies (v2D0) were subtracted from frequencies of postvaccine dose 2 timepoints, including v2D7, v1D10, v1D14, and v2D28. For subjects with missing dose 1 baseline data (2 men and 1 woman), it was assumed that they had no RBD^+^S1^+^ cells at v1D0, which was the case for all remaining 18 subjects with a v1D0 timepoint (except for C1, with a total of two cells across all subjects), so that their other pre–dose 2 timepoints could be included in the analyses. For all other timepoints and non–antigen-specific frequencies, subjects with missing data were excluded from the linear models. *P* values were adjusted with the Benjamini–Hochberg method within each combination of timepoint and antibody endpoint (57). R v3.6.3 was used for this analysis.

### Correlation between Principal Component of Endpoint Antibody Concentrations and Cluster Frequencies.

In addition to modeling of the association of the cluster frequencies to individual antibody concentrations, their relationship to the primary correlated component of the two antibodies to both S-2P and RBD proteins was assessed. PC1 from principal component analysis of the four endpoints (in log2 scale), that is, S-2P IgA/G and RBD IgA/IgG, explained 83.6% of variance across subjects. Associations between PC1 and RBD^+^S1^+^ cluster frequencies at each timepoint were calculated with the same linear models and inclusion criteria described above. The same analysis was carried out for all antigen-nonspecific clusters (cell counts as a fraction of total CD19^+^ B cells) with the baseline prevaccination timepoint (v1D0) included.

## Supplementary Material

Supplementary File

## Data Availability

All data are available in the main text, in online supplemental material, or deposited in Zenodo: doi:10.5281/zenodo.5730466. All codes will be made available upon publication without restrictions.

## References

[1] N. Chaudhary, D. Weissman, K. A. Whitehead, mRNA vaccines for infectious diseases: Principles, delivery and clinical translation. Nat. Rev. Drug Discov. 20, 817–838 (2021). 34433919 10.1038/s41573-021-00283-5 PMC8386155

[2] D. Wrapp , Cryo-EM structure of the 2019-nCoV spike in the prefusion conformation. Science 367, 1260–1263 (2020). 32075877 10.1126/science.abb2507 PMC7164637

[3] F. P. Polack ; C4591001 Clinical Trial Group, Safety and efficacy of the BNT162b2 mRNA covid-19 vaccine. N. Engl. J. Med. 383, 2603–2615 (2020). 33301246 10.1056/NEJMoa2034577 PMC7745181

[4] L. R. Baden ; COVE Study Group, Efficacy and safety of the mRNA-1273 SARS-CoV-2 vaccine. N. Engl. J. Med. 384, 403–416 (2021). 33378609 10.1056/NEJMoa2035389 PMC7787219

[5] R. R. Goel , Distinct antibody and memory B cell responses in SARS-CoV-2 naïve and recovered individuals following mRNA vaccination. Sci. Immunol. 6, eabi6950 (2021). 33858945 10.1126/sciimmunol.abi6950 PMC8158969

[6] D. A. Collier ; CITIID-NIHR BioResource COVID-19 Collaboration, Age-related immune response heterogeneity to SARS-CoV-2 vaccine BNT162b2. Nature 596, 417–422 (2021). 34192737 10.1038/s41586-021-03739-1 PMC8373615

[7] B. J. Boyarsky , Antibody response to 2-dose SARS-CoV-2 mRNA vaccine series in solid organ transplant recipients. JAMA 325, 2204–2206 (2021). 33950155 10.1001/jama.2021.7489 PMC8100911

[8] D. Tsapepas, K. Paget, S. Mohan, D. J. Cohen, S. A. Husain, Clinically significant COVID-19 following SARS-CoV-2 vaccination in kidney transplant recipients. Am. J. Kidney Dis. 78, 314–317 (2021). 34019949 10.1053/j.ajkd.2021.05.004 PMC8129995

[9] B. J. Laidlaw, A. H. Ellebedy, The germinal centre B cell response to SARS-CoV-2. Nat. Rev. Immunol. 22, 7–18 (2022). 34873279 10.1038/s41577-021-00657-1 PMC8647067

[10] R. R. Goel ; UPenn COVID Processing Unit‡, mRNA vaccines induce durable immune memory to SARS-CoV-2 and variants of concern. Science 374, abm0829 (2021). 34648302 10.1126/science.abm0829 PMC9284784

[11] R. A. Elsner, M. J. Shlomchik, Germinal center and extrafollicular B cell responses in vaccination, immunity, and autoimmunity. Immunity 53, 1136–1150 (2020). 33326765 10.1016/j.immuni.2020.11.006 PMC7748291

[12] M. J. Robinson, R. H. Webster, D. M. Tarlinton, How intrinsic and extrinsic regulators of plasma cell survival might intersect for durable humoral immunity. Immunol. Rev. 296, 87–103 (2020). 32592168 10.1111/imr.12895

[13] Y. Kotliarov , Broad immune activation underlies shared set point signatures for vaccine responsiveness in healthy individuals and disease activity in patients with lupus. Nat. Med. 26, 618–629 (2020). 32094927 10.1038/s41591-020-0769-8 PMC8392163

[14] G. M. Li , Pandemic H1N1 influenza vaccine induces a recall response in humans that favors broadly cross-reactive memory B cells. Proc. Natl. Acad. Sci. U.S.A. 109, 9047–9052 (2012). 22615367 10.1073/pnas.1118979109 PMC3384143

[15] J. Wrammert , Rapid cloning of high-affinity human monoclonal antibodies against influenza virus. Nature 453, 667–671 (2008). 18449194 10.1038/nature06890 PMC2515609

[16] J. S. Tsang, Utilizing population variation, vaccination, and systems biology to study human immunology. Trends Immunol. 36, 479–493 (2015). 26187853 10.1016/j.it.2015.06.005 PMC4979540

[17] J. S. Tsang ; Baylor HIPC Center; CHI Consortium, Global analyses of human immune variation reveal baseline predictors of postvaccination responses. Cell 157, 499–513 (2014). 24725414 10.1016/j.cell.2014.03.031 PMC4139290

[18] A. H. Ellebedy , Defining antigen-specific plasmablast and memory B cell subsets in human blood after viral infection or vaccination. Nat. Immunol. 17, 1226–1234 (2016). 27525369 10.1038/ni.3533 PMC5054979

[19] J. S. Turner , SARS-CoV-2 mRNA vaccines induce persistent human germinal centre responses. Nature 596, 109–113 (2021). 34182569 10.1038/s41586-021-03738-2 PMC8935394

[20] W. Kim , Germinal centre-driven maturation of B cell response to mRNA vaccination. Nature 604, 141–145 (2022). 35168246 10.1038/s41586-022-04527-1 PMC9204750

[21] K. Röltgen, S. D. Boyd, Antibody and B cell responses to SARS-CoV-2 infection and vaccination. Cell Host Microbe 29, 1063–1075 (2021). 34174992 10.1016/j.chom.2021.06.009 PMC8233571

[22] A. Pegu ; mRNA-1273 Study Group§, Durability of mRNA-1273 vaccine-induced antibodies against SARS-CoV-2 variants. Science 373, 1372–1377 (2021). 34385356 10.1126/science.abj4176 PMC8691522

[23] L. Stamatatos , mRNA vaccination boosts cross-variant neutralizing antibodies elicited by SARS-CoV-2 infection. Science eabg9175 (2021). 33766944 10.1126/science.abg9175 PMC8139425

[24] J. M. Dan , Immunological memory to SARS-CoV-2 assessed for up to 8 months after infection. Science 371, eabf4063 (2021). 33408181 10.1126/science.abf4063 PMC7919858

[25] C. Gaebler , Evolution of antibody immunity to SARS-CoV-2. Nature 591, 639–644 (2021). 33461210 10.1038/s41586-021-03207-w PMC8221082

[26] A. Sokal , Maturation and persistence of the anti-SARS-CoV-2 memory B cell response. Cell 184, 1201–1213.e14 (2021). 33571429 10.1016/j.cell.2021.01.050 PMC7994111

[27] D. R. Glass , An integrated multi-omic single-cell atlas of human B cell identity. Immunity 53, 217–232.e5 (2020). 32668225 10.1016/j.immuni.2020.06.013 PMC7369630

[28] I. Sanz , Challenges and opportunities for consistent classification of human B cell and plasma cell populations. Front. Immunol. 10, 2458 (2019). 31681331 10.3389/fimmu.2019.02458 PMC6813733

[29] N. Baumgarth, The shaping of a B cell pool maximally responsive to infections. Annu. Rev. Immunol. 39, 103–129 (2021). 33472004 10.1146/annurev-immunol-042718-041238 PMC12203416

[30] K. A. Pape , High-affinity memory B cells induced by SARS-CoV-2 infection produce more plasmablasts and atypical memory B cells than those primed by mRNA vaccines. Cell Rep. 37, 109823 (2021). 34610291 10.1016/j.celrep.2021.109823 PMC8463313

[31] L. B. Rodda , Functional SARS-CoV-2-specific immune memory persists after mild COVID-19. Cell 184, 169–183.e17 (2021). 33296701 10.1016/j.cell.2020.11.029 PMC7682481

[32] M. C. Woodruff , Extrafollicular B cell responses correlate with neutralizing antibodies and morbidity in COVID-19. Nat. Immunol. 21, 1506–1516 (2020). 33028979 10.1038/s41590-020-00814-z PMC7739702

[33] C. Wehr , A new CD21low B cell population in the peripheral blood of patients with SLE. Clin. Immunol. 113, 161–171 (2004). 15451473 10.1016/j.clim.2004.05.010

[34] P. S. Arunachalam , Systems vaccinology of the BNT162b2 mRNA vaccine in humans. Nature 596, 410–416 (2021). 34252919 10.1038/s41586-021-03791-x PMC8761119

[35] M. A. Berkowska , Human memory B cells originate from three distinct germinal center-dependent and -independent maturation pathways. Blood 118, 2150–2158 (2011). 21690558 10.1182/blood-2011-04-345579 PMC3342861

[36] F. Horns , Lineage tracing of human B cells reveals the in vivo landscape of human antibody class switching. eLife 5, e16578 (2016). 27481325 10.7554/eLife.16578 PMC4970870

[37] P. Deepak , Effect of immunosuppression on the immunogenicity of mRNA vaccines to SARS-CoV-2: A prospective cohort study. Ann. Intern. Med. 174, 1572–1585 (2021). 34461029 10.7326/M21-1757 PMC8407518

[38] E. G. Levin , Waning immune humoral response to BNT162b2 Covid-19 vaccine over 6 months. N. Engl. J. Med. 385, e84 (2021). 34614326 10.1056/NEJMoa2114583 PMC8522797

[39] L. Kuri-Cervantes , Comprehensive mapping of immune perturbations associated with severe COVID-19. Sci. Immunol. 5, eabd7114 (2020). 32669287 10.1126/sciimmunol.abd7114 PMC7402634

[40] A. Sette, S. Crotty, Adaptive immunity to SARS-CoV-2 and COVID-19. Cell 184, 861–880 (2021). 33497610 10.1016/j.cell.2021.01.007 PMC7803150

[41] S. F. Andrews , Activation dynamics and immunoglobulin evolution of pre-existing and newly generated human memory B cell responses to influenza hemagglutinin. Immunity 51, 398–410.e5 (2019). 31350180 10.1016/j.immuni.2019.06.024

[42] S. A. Jenks, K. S. Cashman, M. C. Woodruff, F. E. Lee, I. Sanz, Extrafollicular responses in humans and SLE. Immunol. Rev. 288, 136–148 (2019). 30874345 10.1111/imr.12741 PMC6422038

[43] D. Mathew ; UPenn COVID Processing Unit, Deep immune profiling of COVID-19 patients reveals distinct immunotypes with therapeutic implications. Science 369, eabc8511 (2020). 32669297 10.1126/science.abc8511 PMC7402624

[44] J. W. Austin , Overexpression of T-bet in HIV infection is associated with accumulation of B cells outside germinal centers and poor affinity maturation. Sci. Transl. Med. 11, eaax0904 (2019). 31776286 10.1126/scitranslmed.aax0904 PMC7479651

[45] S. A. Jenks , Distinct effector B cells induced by unregulated toll-like receptor 7 contribute to pathogenic responses in systemic lupus erythematosus. Immunity 49, 725–739.e6 (2018). 30314758 10.1016/j.immuni.2018.08.015 PMC6217820

[46] J. J. Knox, A. Myles, M. P. Cancro, T-bet^+^ memory B cells: Generation, function, and fate. Immunol. Rev. 288, 149–160 (2019). 30874358 10.1111/imr.12736 PMC6626622

[47] A. Cho , Anti-SARS-CoV-2 receptor-binding domain antibody evolution after mRNA vaccination. Nature 600, 517–522 (2021). 34619745 10.1038/s41586-021-04060-7 PMC8674133

[48] W. E. Purtha, T. F. Tedder, S. Johnson, D. Bhattacharya, M. S. Diamond, Memory B cells, but not long-lived plasma cells, possess antigen specificities for viral escape mutants. J. Exp. Med. 208, 2599–2606 (2011). 22162833 10.1084/jem.20110740 PMC3244041

[49] R. P. Payne ; PITCH Consortium, Immunogenicity of standard and extended dosing intervals of BNT162b2 mRNA vaccine. Cell 184, 5699–5714.e11 (2021). 34735795 10.1016/j.cell.2021.10.011 PMC8519781

[50] A. Majdoubi , A majority of uninfected adults show preexisting antibody reactivity against SARS-CoV-2. JCI Insight 6, e146316 (2021). 10.1172/jci.insight.146316 PMC8119195 33720905

[51] M. Gu , One dose of COVID-19 nanoparticle vaccine REVC-128 protects against SARS-CoV-2 challenge at two weeks post-immunization. Emerg. Microbes Infect. 10, 2016–2029 (2021). 34651563 10.1080/22221751.2021.1994354 PMC8567933

[52] C. M. Buckner, L. Kardava, S. Moir, Evaluation of B cell function in patients with HIV. Curr. Protoc. Immunol. 10.1002/0471142735.im1213s100 (2013). 23392635

[53] C. M. Buckner , Maintenance of HIV-specific memory B-cell responses in elite controllers despite low viral burdens. J. Infect. Dis. 214, 390–398 (2016). 27122593 10.1093/infdis/jiw163 PMC4936645

[54] S. Van Gassen , FlowSOM: Using self-organizing maps for visualization and interpretation of cytometry data. Cytometry A 87, 636–645 (2015). 25573116 10.1002/cyto.a.22625

[55] E. Becht ., Dimensionality reduction for visualizing single-cell data using UMAP. Nat. Biotechnol. 37, 38–44 (2019). 10.1038/nbt.4314 30531897

[56] A. Kuznetsova, P. B. Brockhoff, R. H. B. Christensen, lmerTest package: Tests in linear mixed effects models. J. Stat. Softw. 82, 1–26 (2017).

[57] Y. Benjamini, Y. Hochberg, Controlling the false discovery rate—A practical and powerful approach to multiple testing. J Roy Stat Soc B Met 57, 289–300 (1995).

[58] S. Moir, A. S. Fauci, B-cell responses to HIV infection. Immunol. Rev. 275, 33–48 (2017). 28133792 10.1111/imr.12502 PMC5300048

[59] S. Portugal, N. Obeng-Adjei, S. Moir, P. D. Crompton, S. K. Pierce, Atypical memory B cells in human chronic infectious diseases: An interim report. Cell. Immunol. 321, 18–25 (2017). 28735813 10.1016/j.cellimm.2017.07.003 PMC5732066

