# Supplementary material for: Early human B cell signatures of the primary antibody response to mRNA vaccination
Source: Proc Natl Acad Sci U S A. 2022 Jun 27;119(28):e2204607119. doi: 10.1073/pnas.2204607119 (PMC9282446; doi:10.1073/pnas.2204607119)
Supplement: Supplementary File [file pnas.2204607119.sapp.pdf]

**Supplementary Information for:**
Early human B cell signatures of the primary antibody response to mRNA vaccination by Kardava et al.

**This PDF file includes:**
Figs. S1 to S3
Tables S1 to S3

# Figure S1

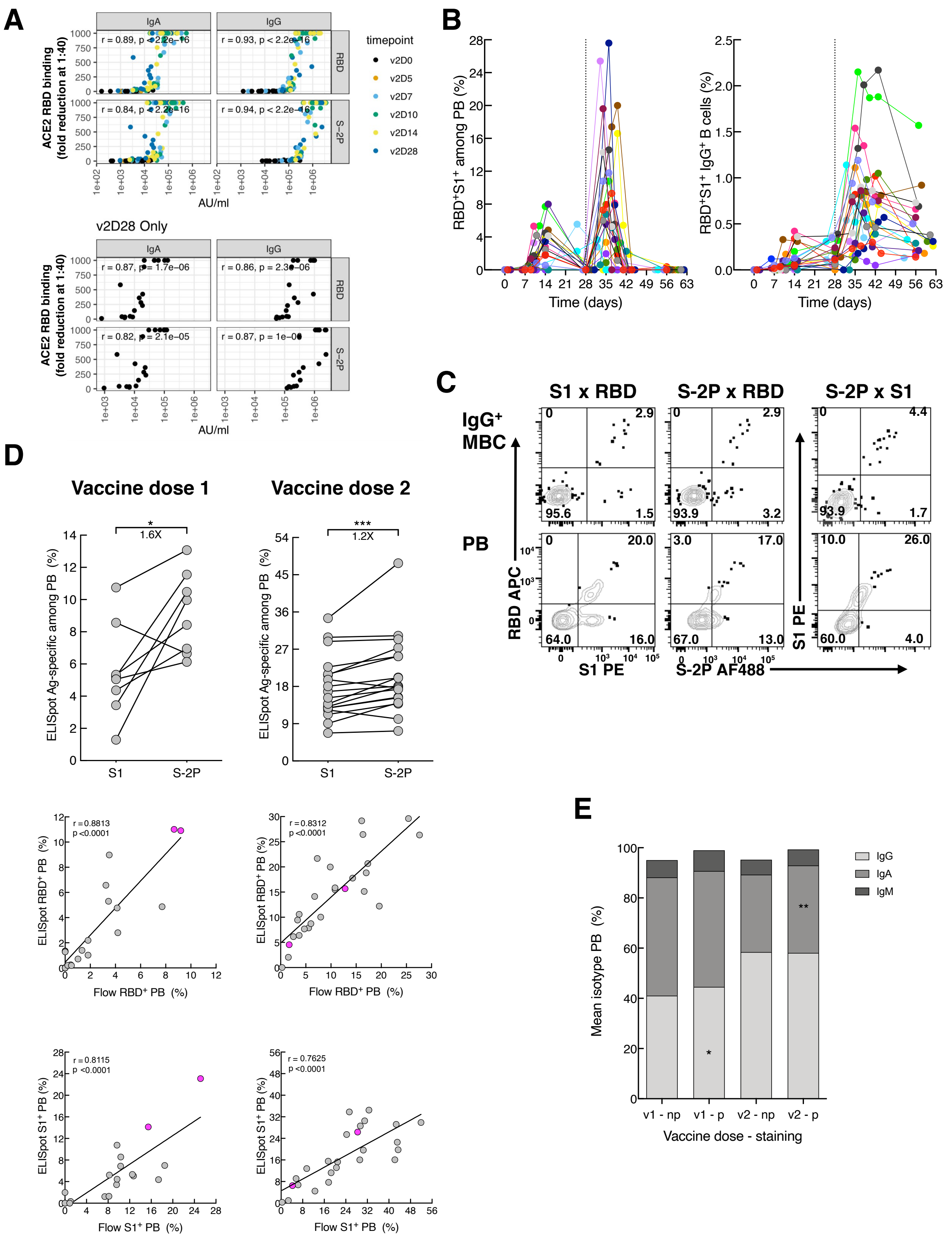
A
IgA
IgG
RBD
S–2P
timepoint
v2D0
v2D5
v2D7
v2D10
v2D14
v2D28
ACE2 RBD binding (fold reduction at 1:40)
AU/ml
v2D28 Only
B
RBD+S1+ among PB (%)
RBD+S1+ IgG+ B cells (%)
Time (days)
C
S1 x RBD
S-2P x RBD
S-2P x S1
IgG+ MBC
PB
RBD APC
S1 PE
S-2P AF488
D
Vaccine dose 1
Vaccine dose 2
ELISpot Ag-specific among PB (%)
S1
S-2P
1.6X
1.2X
ELISpot RBD+ PB (%)
Flow RBD+ PB (%)
ELISpot S1+ PB (%)
Flow S1+ PB (%)
E
Mean isotype PB (%)
IgG
IgA
IgM
v1 - np
v1 - p
v2 - np
v2 - p
Vaccine dose - staining

**Fig. S1.** Antibody and cellular assays with validations and correlations. (A) Correlations between serum IgG and IgA binding to RBD (Fig. 1B) and ACE2 inhibition assay (Fig. 1E) with v2 timepoints shown with color-coding (top panel) or exclusively measurements at v2D28 (lower panel). (B) Longitudinal frequencies of $RBD^+S1^+$ PB and $IgG^+$ B cells vaccinees (*n* = 21) color-coded as in Fig. 1. (C) Binding by flow cytometry of S-2P, S1 and RBD tetramers to PB and $IgG^+$ B cells of an individual at v2D6. (D) Frequencies of ELISpot $S1^+$ versus $S\text{-}2P^+$ PB and correlation between flow cytometric and ELISpot assay-based frequencies of $RBD^+$ and $S1^+$ PB at peak dose 1 and 2 post mRNA-1273 vaccination. (E) Average PB isotype distribution measured by flow cytometry in presence or absence of permeabilization for donors/dose timepoints in (D). Asterisks refer to p values. Donors (D and E) were participants in protocol NCT00001281 (*n* = 20) and NCT04411147 (*n* = 6). Of the 26 donors, two had prior SARS-CoV-2 infection (magenta circles in (D)). Spearman's rank correlation (A and D). Paired t test.*, $p < 0.05$; **, $p < 0.01$ (E) and Wilcoxon signed rank test; ***, $p < 0.001$ (D top panels). AU, arbitrary units; D, day; NP, not permeabilized; P, permeabilized; PB, plasmablasts; RBD, receptor binding domain; S1, spike subunit 1; S-2P, stabilized spike trimer; v, vaccine dose; V, vaccinees.

**Figure S2**

**A**

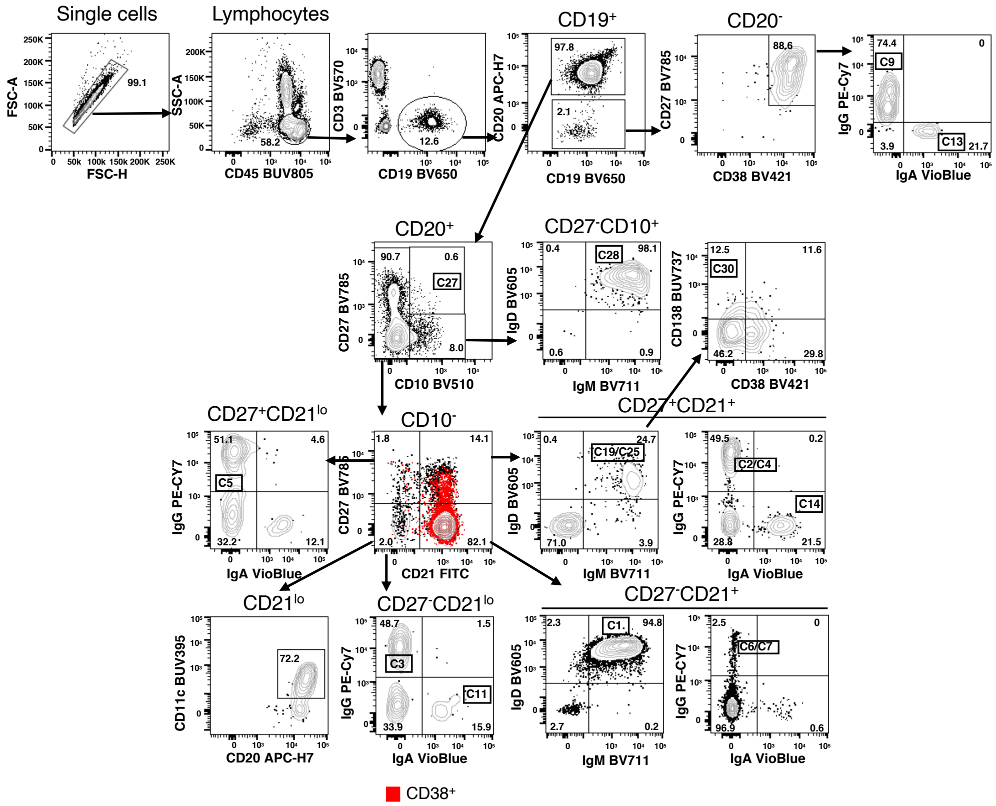
Single cells
Lymphocytes
CD19⁺
CD20⁻
CD20⁺
CD27⁻CD10⁺
CD27⁺CD21lo
CD10⁻
CD27⁺CD21⁺
CD21lo
CD27⁻CD21lo
CD27⁻CD21⁺
FSC-A
FSC-H
SSC-A
CD45 BUV805
CD3 BV570
CD19 BV650
CD20 APC-H7
CD27 BV785
CD38 BV421
IgG PE-Cy7
IgA VioBlue
CD10 BV510
IgD BV605
IgM BV711
CD138 BUV737
CD21 FITC
CD11c BUV395
99.1
58.2
12.6
97.8
2.1
88.6
74.4
C9
C13
3.9
21.7
90.7
0.6
C27
8.0
0.4
C28
98.1
0.6
0.9
12.5
C30
11.6
46.2
29.8
51.1
4.6
C5
32.2
12.1
1.8
14.1
2.0
82.1
C19/C25
24.7
71.0
3.9
49.5
C2/C4
0.2
C14
28.8
21.5
72.2
48.7
1.5
C3
C11
33.9
15.9
2.3
94.8
2.7
0.2
2.5
C6/C7
96.9
0.6
CD38⁺

**B**

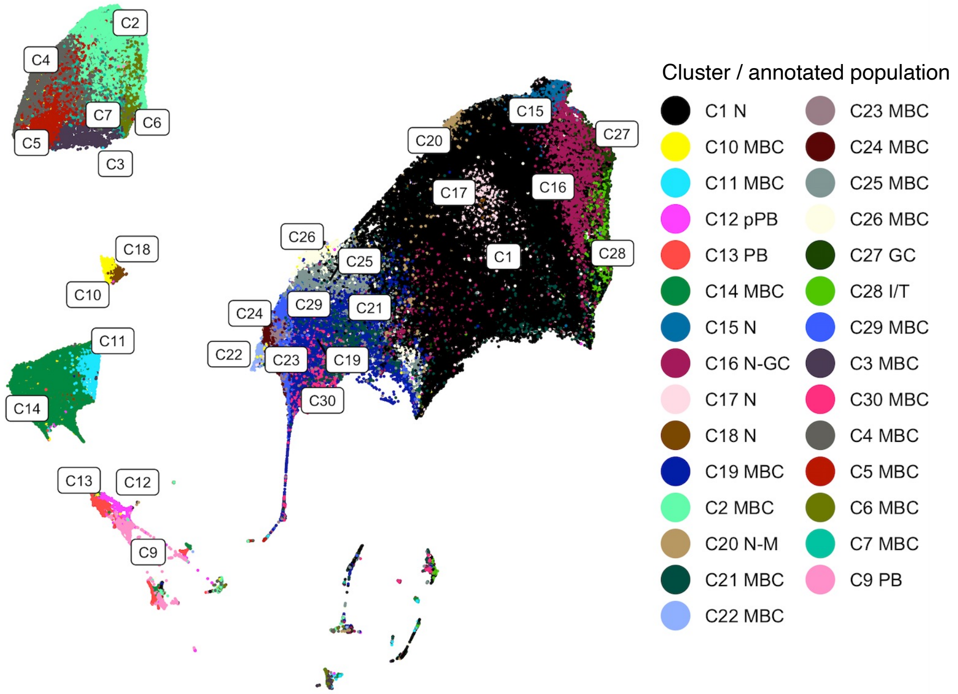
C2
C4
C7
C6
C5
C3
C18
C10
C11
C14
C13
C12
C9
C15
C27
C20
C17
C16
C26
C25
C1
C28
C24
C29
C21
C22
C23
C19
C30
Cluster / annotated population
C1 N
C10 MBC
C11 MBC
C12 pPB
C13 PB
C14 MBC
C15 N
C16 N-GC
C17 N
C18 N
C19 MBC
C2 MBC
C20 N-M
C21 MBC
C22 MBC
C23 MBC
C24 MBC
C25 MBC
C26 MBC
C27 GC
C28 I/T
C29 MBC
C3 MBC
C30 MBC
C4 MBC
C5 MBC
C6 MBC
C7 MBC
C9 PB

**Fig. S2**. Identification of B cell populations and corresponding clusters. (A) Representative flow cytometry of B cells at v2D7 with gating strategy, corresponding cluster designations and marker distribution. (B) Uniform Manifold Approximation and Projection (UMAP) displaying location of individual cell population clusters, similar to Fig. 2A but displaying annotations of individual clusters.

**Figure S3**

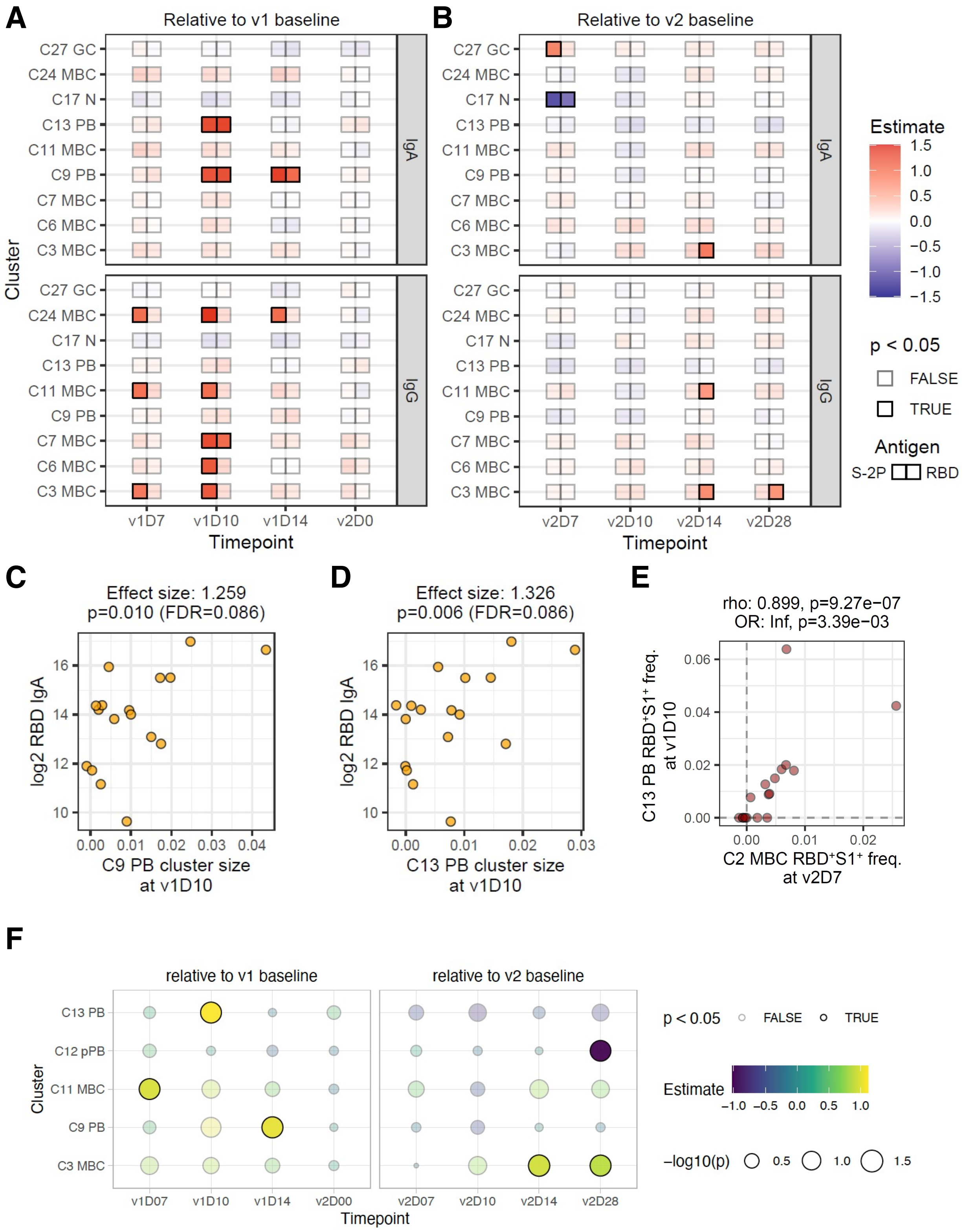
A
Relative to v1 baseline
B
Relative to v2 baseline
C27 GC
C24 MBC
C17 N
C13 PB
C11 MBC
C9 PB
C7 MBC
C6 MBC
C3 MBC
IgA
IgG
Cluster
v1D7
v1D10
v1D14
v2D0
v2D7
v2D10
v2D14
v2D28
Timepoint
Estimate
1.5
1.0
0.5
0.0
−0.5
−1.0
−1.5
p < 0.05
FALSE
TRUE
Antigen
S-2P
RBD
C
Effect size: 1.259
p=0.010 (FDR=0.086)
log2 RBD IgA
C9 PB cluster size at v1D10
D
Effect size: 1.326
p=0.006 (FDR=0.086)
C13 PB cluster size at v1D10
E
rho: 0.899, p=9.27e−07
OR: Inf, p=3.39e−03
C13 PB RBD⁺S1⁺ freq. at v1D10
C2 MBC RBD⁺S1⁺ freq. at v2D7
F
relative to v1 baseline
relative to v2 baseline
C13 PB
C12 pPB
C11 MBC
C9 PB
C3 MBC
Cluster
v1D07
v1D10
v1D14
v2D00
v2D07
v2D10
v2D14
v2D28
Timepoint
p < 0.05
FALSE
TRUE
Estimate
−1.0
−0.5
0.0
0.5
1.0
−log10(p)
0.5
1.0
1.5

**Fig. S3.** Additional associations between cluster frequencies and v2D28 endpoint SARS-CoV-2 antibody titers. (A and B) Similar to Fig. 4A and 4B, but for associations with antigen non-specific cells. (C and D) Similar to Fig. 4C but showing association between endpoint RBD IgA titers and antigen non-specific cells, i.e., cluster size as a fraction of total $CD19^+$ cell counts, within C9 (C), and C13 (D), on v1D10. (E) Correlation between $RBD^+S1^+$ cell frequencies within C2 on v2. (F) Similar to Fig. 4F, associations between the first principal component (PC1 – isotype independent) of antibody endpoint at 28 days after second dose (v2D28) and antigen non-specific cells.

## Table S1: Participant information and visits

| Participant | Age at Vaccination | Gender at Birth | Vaccine Dose 1[#] | | | | Vaccine Dose 2[#] | | | | | | Days Between Doses |
|---|---|---|---|---|---|---|---|---|---|---|---|---|---|
| | | | D0 | D7 | D10 | D14 | D0 | D5 | D7 | D10 | D14 | D28 | |
| VAC-001 | 52 | M | 1 | 7 | 9 | 14 | 0 | | 6 | 9 | 13 | 28 | 28 |
| VAC-002 | 60 | F | 0 | 7 | 11 | 14 | 0 | | 7 | 9 | 14 | 28 | 27 |
| VAC-003 | 61 | F | 0 | 6 | 9 | 14 | 0 | | 6 | 9 | 15 | 28 | 28 |
| VAC-004 | 54 | F | 0 | 7 | 9 | 15 | -1 | | 6 | 9 | 13 | 28 | 28 |
| VAC-005 | 64 | F | 0 | 7 | 11 | 14 | 0 | | 6 | 11 | 14 | 33 | 28 |
| VAC-611 | 51 | F | -21 | 7 | 10 | 14 | 0 | | 7 | 9 | 14 | 28 | 29 |
| VAC-613 | 58 | F | 2 | 7 | 10 | 15 | 0 | | 7 | 10 | 13 | 29 | 28 |
| VAC-638 | 56 | F | 0 | 7 | 10 | 14 | 0 | 5 | 7 | | 14 | 31 | 31 |
| VAC-662 | 54 | M | 0 | | | 15 | 0 | 5 | 7 | 9 | 14 | 28 | 28 |
| VAC-676 | 55 | M | 0 | 8 | | 15 | -1 | | 6 | 10 | 14 | 31 | 29 |
| VAC-683 | 65 | M | 0 | 7 | 10 | 15 | 0 | | 7 | 9 | 14 | 28 | 30 |
| VAC-685 | 44 | M | -1 | 7 | 10 | 14 | 0 | | 7 | 10 | 14 | 28 | 28 |
| VAC-687 | 55 | F | 0 | 8 | 11 | 14 | 0 | | 7 | 11 | 14 | 28 | 28 |
| VAC-713* | 44 | M | 1 | 6 | | 14 | | | | | | | |
| VAC-715 | 44 | F | 0 | 7 | | | 0 | | 7 | 10 | 14 | 28 | 25 |
| VAC-716 | 41 | F | 0 | 7 | 12 | 14 | 0 | | 7 | 11 | 14 | 28 | 29 |
| VAC-717 | 60 | F | | 7 | 11 | 14 | 0 | 5 | 7 | 9 | | 28 | 34 |
| VAC-718 | 40 | F | -1 | 7 | 11 | | 0 | | 7 | 11 | 13 | 31 | 28 |
| VAC-719 | 56 | M | | 7 | 11 | 14 | 0 | | 7 | 11 | 14 | 28 | 28 |
| VAC-720 | 36 | M | 0 | 7 | 10 | 14 | 0 | | 6 | 11 | 14 | 28 | 27 |
| VAC-721 | 65 | M | | 7 | 10 | 15 | -1 | | 6 | 10 | 12 | 26 | 26 |
| **Median[†]/N** | **55[†]** | **9M/12F** | **18** | **20** | **17** | **19** | **20** | **3** | **20** | **19** | **19** | **20** | **28[†]** |

[*]Lost to follow-up after contracting COVID-19 after dose 1

[#]Numbers indicate actual date of visit relative to vaccination day

## Table S2: 17-color flow cytometry panel

| Reagent | Clone | Source | Catalogue Number | Dilution |
|---|---|---|---|---|
| Mouse anti-human CD11c BUV395 | B-ly6 | BD Biosciences | 563787 | 1:100 |
| Mouse anti-human CD138 BUV737 | MI15 | BD Biosciences | 612834 | 1:200 |
| Mouse anti-human CD45 BUV805 | HI30 | BD Biosciences | 612891 | 1:100 |
| Mouse anti-human CD38 BV421 | HIT2 | BD Biosciences | 562444 | 1:100 |
| Mouse anti-human CD19 BV650 | SJ25-C1 | BD Biosciences | 563226 | 1:100 |
| Mouse anti-human CD10 BV510 | HI10a | BD Biosciences | 563032 | 1:100 |
| Mouse anti-human CD3 BV570 | UCHT1 | Biolegend | 300436 | 1:100 |
| Mouse anti-human IgD BV605 | IA6–2 | Biolegend | 348232 | 1:100 |
| Mouse anti-human IgM BV711 | MHM-88 | Biolegend | 314540 | 1:100 |
| Mouse anti-human CD14 BV750 | 63D3 | Biolegend | 367136 | 1:100 |
| Mouse anti-human CD27 BV785 | O323 | Biolegend | 302832 | 1:100 |
| Mouse anti-human CD21 FITC | BU32 | Biolegend | 354910 | 1:100 |
| Mouse anti-human IgG PE-Cy7 | G18–145 | BD Biosciences | 561298 | 1:100 |
| Mouse anti-human CD20 APC-H7 | 2H7 | BD Biosciences | 560734 | 1:100 |
| Mouse anti-human IgA VioBlue | IS11-8E10 | Miltenyi Biotec | 130-113-479 | 1:200 |
| SARS-CoV-2 S protein S1 | | Biolegend | 793806 | |
| SARS-CoV-2 S protein RBD | | In-house | | |

**Table S3: Detailed cluster annotations and designations**

| Cluster | Population | Ig isotype | Defining markers | Designations/other names* |
|---|---|---|---|---|
| 28 | I/T | IgM>D | $CD10^{+}CD38^{++}CD27^{-}CD138^{+}$ | |
| 16 | N-GC | IgD>M | $CD10^{+}CD38^{+}CD27^{lo}$ | |
| 27 | GC | IgM>D | $CD10^{+}CD38^{++}CD27^{+}CD138^{lo}$ | GC founder |
| 23 | MBC | IgD>M | $CD27^{lo}CD38^{-}CD20^{++}CD21^{lo}CD11c^{+}$ | Nonconventional MBC/Atypical/TLM |
| 29 | MBC | IgM>D | $CD27^{+}CD38^{-}CD20^{++}CD21^{lo}CD11c^{+}$ | Nonconventional MBC/Atypical/AM |
| 30 | MBC | IgM>D | $CD27^{+}CD38^{-}CD138^{+}$ | Conventional MBC |
| 21 | N | IgD=M | $CD38^{-}$ | |
| 19 | MBC | IgM>D | $CD27^{+}CD38^{-}$ | Conventional MBC |
| 25 | MBC | IgM>D | $CD27^{+}CD38^{+}$ | Conventional MBC |
| 22 | MBC | $IgD^{+}IgM^{-}$ | $CD27^{+}CD38^{-}$ | Conventional MBC/IgD only MBC |
| 24 | MBC | IgD>M | $CD27^{lo}CD38^{-}CD20^{++}CD21^{lo}CD11c^{+}$ | Nonconventional MBC/Atypical/TLM |
| 15 | MBC | IgD>M | $CD27^{lo}CD38^{+}CD11c^{+}$ | Nonconventional MBC |
| 20 | N-MBC | IgD>M; IgG | $CD27^{lo}CD38^{+}$ | Likely bound IgG |
| 1 | N | IgD>M | $CD38^{+}$ | |
| 17 | N | $IgD^{+}IgM^{-}$ | $CD38^{+}$ | |
| 12 | pPB | IgA | $CD27^{lo}CD38^{++}CD20^{-}CD21^{lo}$ | |
| 13 | PB | IgA | $CD27^{+}CD38^{+++}CD20^{-}CD21^{lo}$ | |
| 9 | PB | IgG | $CD27^{+}CD38^{+++}CD20^{-}CD21^{lo}$ | |
| 8 | NB | Multiple Ig | $CD10^{+}CD14^{lo}$ | Granulocyte |
| 5 | MBC | IgG | $CD27^{+}CD38^{-}CD20^{++}CD21^{lo}CD11c^{+}$ | Nonconventional MBC/Atypical/AM |
| 3 | MBC | IgG | $CD27^{-}CD38^{-}CD20^{++}CD21^{lo}CD11c^{+}$ | Nonconventional MBC/Atypical/TLM/DN2 |
| 4 | MBC | IgG | $CD27^{+}CD38^{-}$ | Conventional MBC |
| 2 | MBC | IgG | $CD27^{+}CD38^{+}$ | Conventional MBC |
| 7 | MBC | IgG | $CD27^{-}CD38^{-}$ | Nonconventional MBC |
| 6 | MBC | IgG | $CD27^{-}CD38^{+}$ | Nonconventional MBC |
| 11 | MBC | IgA | $CD27^{lo}CD38^{-}CD20^{++}CD21^{-}CD11c^{+}$ | Nonconventional MBC/Atypical/TLM/DN2 |
| 14 | MBC | IgA | $CD27^{+}CD38^{+}$ | Conventional MBC |
| 26 | MBC | $IgM^{+}IgD^{-}$ | $CD27^{+}CD38^{+}$ | Conventional MBC/IgM only memory |
| 18 | | Low multiple Ig | $CD38^{+}$ | |
| 10 | MBC | Low multiple Ig | $CD27^{+}CD38^{lo}$ | Conventional MBC |

AM, activated memory; DN2 double negative 2; GC, germinal center; I/T immature/transitional; MBC, memory B cell; N, naïve; NB, not B cell; PB, plasmablast; pPB, pre-PB; TLM, tissue-like memory

*References for designations: (42, 58, 59)
